# *Beauveria bassiana* In Vitro Compatibility with Pesticides: A Mixture Efficacy Prediction against the Sugar Cane Weevil, *Sphenophorus levis*

**DOI:** 10.1021/acsomega.5c05193

**Published:** 2025-07-21

**Authors:** Ricardo Antonio Polanczyk, Giovani Smaniotto, Kelly Cristina Gonçalves

**Affiliations:** Department of Plant Protection, Faculty of Agricultural and Veterinary Sciences, 28108 Universidade Estadual Paulista Júlio de Mesquita Filho, Jaboticabal, São Paulo 14884-900, Brazil

## Abstract

In vitro compatibility assays are required to identify incompatible tank mixtures. The selected *Beauveria bassiana* strain IBCB 170 and 11 pesticides registered against *Shenophorus levis* (half, complete, and double doses) were added to PDA medium. After incubation, the colonies were measured using ImageJ software, and conidial counting and germination were obtained using a light microscope. The data were analyzed using Tukey’s 5% test, and the degree of compatibility was determined using a biological index (BI). All mixtures reduced vegetative growth, except for DiamanteSC and AltacorWG, at full and double doses. Singular BRSC and DiamanteSC produced conidia similar to those of the control. DiamanteSC, Imidacloprid Nortox 480SC, and Regent 800WG showed similar viability to the control at half the dose. Indeed, SingularBR SC is compatible with full and double doses, whereas DiamanteSC is only compatible with full doses. These data highlight the need for compatibility laboratory evaluation before field application.

## Graphical abstract

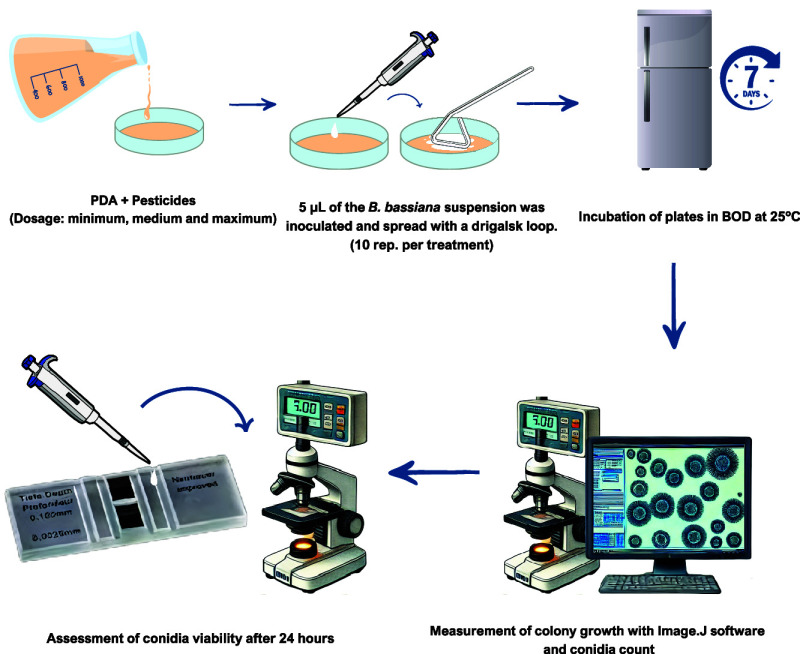

## Introduction

Operating costs directly impact financial returns, so the farmer uses tank-mixing primarily to optimize farming operations, such as to increase the spectrum of spraying, which simultaneously allows the treatment of multiple targets.^1–3^ It is important to point out the ‘multiple attack’ approach can increase control efficiency compared to individual use.^4–6^ With the increased use of biopesticides based on entomopathogenic microorganisms in pest control,^7,8^ it has become common to mix them with pesticides.^9–11^ A pioneering publication on the potential of such products in pest control stated that biopesticides can be used with pesticides to achieve synergistic or additive effects.^12^

Pesticides can affect entomopathogenic microorganisms, resulting in deleterious, null or synergistic effects. These harmful effects include the inhibition of vegetative growth, reproduction, germination, reduced virulence, and pathogenic mutations. Synergistic effects occur when the susceptibility of an arthropod pest is compromised by a certain level of abiotic or biotic stress and the combined action of pesticides and microorganisms promotes an increase in host susceptibility.^12,13^

However, it should be noted that the large number of pesticides combined in the tank makes it impossible to predict how the components of the mixture will interact and that the additive or synergistic effect of the mixture depends on the mixed components.^14–16^ The antagonism between the components of the mixture may lead to reduced application efficiency, resulting in increased production costs and environmental impacts owing to the need for reapplication. One method to predict compatibility is to conduct in vitro assays to predict further field incompatibility tests.^17–22^

Because of the reduced field efficacy of registered pesticides against *Sphenophorus levis* (Coleoptera: Curculionidae), the most important pest of Brazilian sugar cane,^23,24^ and the need for eco-friendly strategies, such as entomopathogenic fungi, to reduce pesticide use and preserve ecosystem services,^25^ we conducted this study using pesticides and *Beauveria bassiana*.

## Material and Methods

The *B. bassiana* strain IBCB 170, previously selected against *S. levis*,^26^ was obtained from the isolate bank of the Biological Control Laboratory of the Central Experimental Center of the Biological Institute in Campinas, Oldemar Cardim Abreu. It was multiplied in Potato Dextrose Agar (PDA) culture medium and maintained in an acclimatized chamber at 25 ± 2 °C, 70 ± 10% RH, and a 12:12 h photoperiod. Before the compatibility assays, the fungal viability was 87.5%.

The pesticides listed in Table 1 were chosen based on recommendations for controlling *S. levis*.^27^ Bioassays were performed using the manufacturer’s recommended minimum (half) and maximum (double) doses, as well as the average (full dose), to simulate a wider impact of pesticides on the fungus. The previously described^13,28^ was used to assess colony growth (vegetative growth) and conidial production (sporulation). Potato Dextrose Agar (PDA) culture medium was used, diluting 42 g in one liter of deionized water. After dilution, the medium was autoclaved at 121 °C for 15 min.

**Table 1:** Products Assayed for In Vitro compatibility with *Beauveria bassiana* IBCB 170^a^

			dose		
product	active ingrediente	chemical group	half	full	double
Actara250 WG	tiametoxam	neonicotinoid	0.4 kg ha^–1^	0.7 kg ha^–1^	1.0 kg h^–1^
Altacor WG	chlorantraniliprole	anthranilamide	0.3 kg ha^–1^	0.37 kg ha^–1^	0.4 kg ha^–1^
Capture 400 EC	bifenthrin	pyrethroid	0.3 L ha^–1^	0.45 L ha^–1^	0.6 L ha^–1^
Diamante SC	imidacloprid	neonicotinoid	0.75 L ha^–1^	0.82 Lha^–1^	1.0 L ha^–1^
EngeoPleno SC	tiametoxam + lambda- cyhalothrin	neonicotinoid + pyrethroid	1.5 L ha^–1^	2.0 kg ha^–1^	2.5 kg ha^–1^
Evidence 700 WG	imidacloprid	neonicotinoid	0.2 kg ha^–1^	0.4 kg ha^–1^	0.8 kg ha^–1^
Imidacloprid Nortox 480 SC	imidacloprid	neonicotinoid	0.72 L ha^–1^	0.84 L ha^–1^	0.96 L ha^–1^
MarshalStar EC	carbosulfan	benzofuranyl methylcarbamate	3.5 L ha^–1^	4.0 L ha^–1^	4.5 L ha^–1^
Regent 800 WG	fipronil	phenylpyrazole	0.25 kg ha^–1^	0.37 kg ha^–1^	0.5 kg ha^–1^
SingularBR SC	fipronil	pyrazole	0.3 L ha^–1^	0.5 L ha^–1^	0.7 L ha^–1^
Talisman EC	bifenthrin + carbosulfan	pyrethroid + benzofuranyl methylcarbamate	3.0 L ha^–1^	4.0 L ha^–1^	5.0 L ha^–1^

a SC: suspension concentrate; WG: water dispersible granules; EC: emulsion concentrate.

To mix the pesticides, the medium was heated to 45 °C, which maintained its liquid form without affecting its chemical properties.^29^ After homogenizing the medium with pesticides, 10 mL was poured into autoclaved Petri dishes with a diameter of 9 cm, with 10 replicates for each treatment. Each treatment consisted of a single dose of the pesticide plus the fungus. Once the medium solidified, an aliquot of 5.0 μL of the fungus containing 10^9^ *B. bassiana* conidia was inoculated into the center of the plate. After inoculation, the plates were sealed with PVC plastic and placed in a germination chamber for 7 days at 27 ± 2 °C, with an average relative humidity of 70 ± 10% and a 12:12 h photoperiod.

Subsequently, the number of colonies was counted. All colonies were photographed individually and measured using ImageJ software.^30^ After the measurements, the conidia were counted and the colonies were removed from the culture medium and placed in Falcon tubes containing 10 mL of deionized and autoclaved water. This suspension was homogenized, and a small aliquot was removed to visualize the reproductive structures using a Zeiss optical microscope (Stemi 305) and Neubauer chamber (400×).

The microscope slide technique (26 × 76 mm) was used to determine the germination (viability) of the fungus.^31^ For this test, previously sterilized microscope slides were used. After coating with PDA culture medium for each treatment, the medium solidified and a *B. bassiana* aliquot of 5.0 μL was inoculated. A circle was created to make it easy to identify where the fungus was being inoculated. Each treatment consisted of five replicates.

After 24 h, germinated and nongerminated spores were quantified using an optical microscope (Zeiss) to determine the germination percentage for each treatment. The experiment was conducted using a completely randomized design. The data were subjected to an analysis of variance. The parameters evaluated in each treatment were compared using Tukey’s test at the 5% probability level.

The following formula was used to standardize compatibility (IB = 47 [CV] + 43 [ESP] + 10 [GER]/100): IB = biological index; CV = the percentage of vegetative growth of the colony after 7 days compared to the control; ESP = the percentage of sporulation of the colony after 7 days compared to the control; GER = the rate of germination of conidia after 24 h.^13^

## Results and Discussion

At half the dose, vegetative growth was significantly affected in all treatments, ranging from a 65.5% (DiamanteSC) to 100% (Evidence700 WG) reduction in growth. At full and double doses, Capture 400 EC, MarshalStar EC, Evidence 700 WG, and EngeoPleno SC inhibited the development of the fungus by 100%. At the same time, there was no significant difference between the control and SingularBR SC or Diamante SC, with the latter showing more remarkable numerical growth than the control (Table 2). This phenomenon may be due to some bioremediation characteristics of *B. bassiana*, in which the fungus can use the active ingredient (a.i.) and/or formulation components for growth.^32,33^

**Table 2:** *Beauveria bassiana* IBCB 170 Conidia Viability (%) (±SE) to Pesticides in In Vitro Compatibility Assays^a^

product	half dose	full dose	double dose	*F*_(gl)_	*P*
control	100.0 Aa	100.0 Aa	100.0 Aa	0.00_(2.12)_	>0.0500
Actara250WG	64.4 ± 18.8 BCDa	29.9 ± 3.2 Bab	2.9 ± 1.4 Db	7.77_(2.12)_	0.0068
Altacor WG	48.9 ± 8.9 CDEa	20.0 ± 2.0 Bab	0.0 Db	3.78_(2.12)_	0.0532
Capture 400 EC	38.7 ± 9.3DEFa	21.3 ± 3.9Bab	0.0 Db	11.07_(2.12)_	0.0019
Diamante SC	93.9 ± 0.81 ABa	20.0 ± 20.0 Bb	0.0 Db	18.33_(2.12)_	0.0002
EngeoPleno SC	22.8 ± 7.33 EFGa	0.0 Bb	0.0 Db	9.68_(2,12)_	0.0031
Evidence 700 WG	0.0 Ga	0.0 Ba	0.0 Da	0.00_(2.12)_	>0.0500
Imidacloprid Nortox 480 SC	78.0 ± 3.8 ABCa	0.0 Bb	0.0 Db	402.6_(2.12)_	0.0001
MarshalStar EC	0.0 Ga	0.0 Ba	0.0 Da	0.00_(2.12)_	>0.0500
Regent 800 WG	96.7 ± 1.31 ABa	31.2 ± 4.6 Bb	10.8 ± 3.3 Cc	175.7_(2.12)_	0.0001
SingularBR SC	59.3 ± 6.13 CDa	24.0 ± 14.6 Bb	0.0 Db	10.5_(2.12)_	0.0023
Talisman EC	8.8 ± 2.41 F Ga	2.9 ± 0.8 Bb	0.0 Db	9.1_(2.12)_	0.0038
*F*_(gl)_	29.72_(12.52)_	11.91_(12.52)_	565.82_(12.52)_		
*P*	<0.0001	<0.0001	<0.0001		

a Averages followed by the same uppercase letter in the column and lowercase letter in the row do not differ significantly according to Tukey’s test (*P* < 0.05).

This growth inhibition may be related to the type of active ingredient, its concentration, and presence of coformulants.^34^ Diamante SC had the same concentration (480 g/L i.a.), whereas Evidence 700 WG had a higher concentration (700 g/kg i.a.) of the same active ingredient (Imidacloprid Nortox 480 SC). Another factor influencing the development of the fungus with these pesticides may be related to their formulation: Diamante SC has a suspension concentrate (SC), whereas Evidence 700 WG contains water-dispersible granules (WG). A similar result was observed for SingularBR SC and Regent 800 WG, which have fipronil as their active ingredient, with the concentration (Singular 600 g/L a.i., Regent 800 g/L a.i.) differing according to the type of formulation (Table 1).

Several authors have reported that insecticides containing imidacloprid as an active ingredient do not affect the vegetative growth of *B. bassiana*,^35–37^ similar to the data found in our study with the pesticide Diamante SC. However, the same pesticide, Evidence 700 WG, proved compatible with *Metarhizium rileyi* (Farlow),^11^ demonstrating a difference in interaction according to the fungal species.

Pesticides containing the active ingredients bifenthrin and carbosulfan, or a mixture of both, reduced the vegetative growth of the fungus at all doses (Table 2). Several authors have reported the adverse effects of pyrethroid (Capture 400 EC) on the development of *B. bassiana*.^17,38–40^ These reports are in line with the results of the present study, which showed a negative effect of this de novo class on the fungus.

When the fungus was mixed with EngeoPleno SC, the product negatively affected fungal development as the dose was increased. However, the pesticide had no adverse effects on *B. bassiana* IBCB 276.^35^ This highlights the tremendous genetic variability among isolates of entomopathogenic fungi,^2^ which can affect their interactions with pesticides.

Regarding the number of conidia produced (sporulation), all treatments affected this parameter, except for Actara250 WG, SingularBR SC, and Diamante SC, at all doses tested, with deleterious effects ranging from 8.2 to 100% (Table 3). Increasing the dose implied an increase in the number of treatments that inhibited conidial production, with four pesticides (Talisman EC, Capture 400 EC, MarshalStar EC, and Evidence 700 WG) causing this effect at the double dose.

**Table 3:** *Beauveria bassiana* IBCB 170 Vegetative Growth (cm^2^) (±SE) to Pesticides in In Vitro Compatibility Assays^a^

product	half dose	full dose	double dose	*F*_(gl)_	*P*
control	9.14 ± 2.6 Aa	9.14 ± 2.6 ABa	9.14 ± 2.6 Aa	0.00_(2.27)_	>0.0500
Actara250 WG	1,40 ± 0,1 BCb	3.40 ± 0.8 Da	3.93 ± 0.3 ABa	5.97_(2.27)_	0.0071
Altacor WG	3.14 ± 0.5 BCa	2.52 ± 0.6 CDa	1.86 ± 0.3 CDa	1.45_(2.27)_	0.2523
Capture 400 EC	0.74 ± 0.23 Ca	0.00 D b	0.00 Db	10.22_(2.27)_	0.0005
Diamante SC	3.77 ± 0.72 BCb	11.19 ± 1.8 Aa	9.00 ± 0.64 Aa	10.20_(2.27)_	0.0005
EngeoPleno SC	3.11 ± 0.34 BCa	0.00 D b	0.00 Db	81.43_(2.27)_	0.0001
Evidence 700 WG	0.00 Ca	0.00 D a	0.00 Da	0.00_(2.27)_	>0.0500
Imidacloprid Nortox 480 SC	2.48 ± 0.30 BCab	3.61 ± 0.3 BCa	2.15 ± 0.54 CDb	3.63_(2.27)_	0.0401
MarshalStar EC	0.11 ± 0.03 Ca	0.00 D b	0.00 Db	10.44_(2.27)_	0.0004
Regent 800 WG	3.16 ± 1.02 BCab	3.80 ± 0.9 BCa	0.69 ± 0.27 CDb	3.83_(2.27)_	0.0342
SingularBR SC	3.16 ± 0.49 BCa	7.04 ± 2.3 ABa	7.19 ± 0.45 ABa	2.63_(2.27)_	0.0906
TalismanEC	1.21 ± 0.15 Ca	0.33 ± 0.20 Db	0.00 Db	17.49_(2.27)_	0.0001
*F*_(gl)_	8.21_(12.117)_	9.71_(12.117)_	19.13_(12.117)_		
*P*	0.0001	0.0001	0.0001		

a Averages followed by the same uppercase letter in the column and lowercase letter in the row do not differ significantly according to Tukey’s test (*P* < 0.05).

Pesticides significantly reduced the germination rate of *B. bassiana* (Table 4). Only half of the doses of the three treatments (DiamanteSC, Imidacloprid Nortox 480 SC, and Regent 800 WG) showed values close to 80%, which is considered satisfactory for germination.^40^ At the full dose, the maximum value obtained was 29.9%, whereas at the double dose, germination was almost zero, with only one treatment (Actara250 WG) showing 2.9% germination.

**Table 4:** *Beauveria bassiana* IBCB 170 Produced Conidia (10^7^) (±SE) with Pesticides in In Vitro Compatibility Assays^a^

product	half dose	full dose	double dose	*F*_(gl)_	*P*
control	1.83 ± 0.5 Ba	1.83 ± 0.5 ABa	1.83 ± 0.5 Aa	0.00_(2.27)_	1.0000
Actara250WG	0.84 ± 0.13 Ba	0.62 ± 0.1 CDa	1.34 ± 0.4 ABCa	1.56_(2.27)_	0.2286
Altacor WG	0.39 ± 0.26 Ba	0.51 ± 0.14CDa	0.15 ± 0.02 CDa	1.08_(2.27)_	0.3549
Capture 400 EC	0.37 ± 0.06 Ba	0.01 ± 0.01 Db	0.00 Db	26.96_(2.27)_	0.0001
Diamante SC	1.68 ± 0.4 Ba	0.85 ± 0.2 BCb	0.52 ± 0.1 BCDb	3.68_(2.27)_	0.0387
EngeoPleno SC	0.58 ± 0.11 Ba	0.06 ± 0.03 Db	0.03 ± 0.01 Db	20.22_(2.27)_	0.0001
Evidence 700 WG	0.00 Ba	0.00	0.00 Da	0,00_(2.27)_	1.000
Imidacloprid Nortox 480 SC	0.10 ± 0.01 Ba	0.13 ± 0.02 Da	0.11 ± 0.01 CDa	1.2_(2.27)_	0.3178
MarshalStar EC	0.03 ± 0.01 Ba	0.00 Db	0.00 Db	6.85_(2.27)_	0.0039
Regent 800 WG	0.48 ± 0.13 Ba	0.19 ± 0.06 Dab	0.09 ± 0.04 Db	5.58_(2.27)_	0.0094
SingularBRSC	1.28 ± 0.19 Ba	1.37 ± 0.2 BCa	1.53 ± 0.50 ABa	0.13_(2.27)_	0.8761
Talisman EC	0.58 ± 0.13 Ba	0.04 ± 0.01 Db	0.00 Db	16.73_(2.27)_	0.0001
*F*_(gl)_	28.15_(12.117)_	13.61_(12.117)_	7.74_(12.117)_		
*P*	0.0001	0.0001	0.0001		

a Averages followed by the same uppercase letter in the column and lowercase letter in the row do not differ significantly according to Tukey’s test (*P* < 0.05).

The methodology used to determine the viability of conidia proposes that evaluations should be carried out 24 h after contact between the fungus and pesticide. The viability data (Table 2) showed that most of the pesticides, mainly at the double dose, did not allow the fungus to germinate, but colony development was reported (Table 3), and consequently, conidia production (Table 4). This may be related to the adaptation of the fungus to the substrate (medium or pesticide). Another observation was that the fungus initiated the germination process. However, no colony formation or sporulation was observed due to the insecticide.

The conidial germination process is more critical than vegetative growth and sporulation, as this is the first stage to trigger epizootic.^2^ Conidia are responsible for the reproduction and dissemination of fungi under field conditions; that is, the fungus depends on this process to successfully infect the host.^41–43^ Therefore, when a pesticide causes a significant decrease in spore germination, it can reduce the effectiveness of the entomopathogen on its target. However, vegetative growth and sporulation are of great importance in the process of secondary infections caused by fungi; therefore, they must be considered for the virulence and persistence of the fungus in the environment.^43^

According to the BI index (Table 5), all pesticides were toxic to *B. bassiana* at half the dose, whereas SingularBR SC was moderately toxic. At full dose, only SingularBR SC and Diamante SC were compatible, whereas at double dose, only SingularBRSC was compatible, and Diamante SC was moderately toxic. Pesticides classified as moderately toxic can be used at lower doses than those assayed here to favor compatibility with *B. bassiana* under field conditions. In this case, it is essential to note the necessity of rotating the active ingredients to delay resistance evolution.^44^

**Table 5:** Biological Index (BI) and Compatibility Classification (CC) in the Interaction between *Beauveria bassiana* IBCB 170 and Pesticides^a^

	half dose		full dose		double dose	
product	BI	CC	BI	CC	BI	CC
Actara250 WG	33.35	T	35.00	T	53.22	MT
Altacor WG	31.14	T	26.86	T	12.67	T
Capture 400 EC	16.39	T	2.45	T	0.0	T
Diamante SC	68.29	T	73.53	C	55.77	MT
EngeoPleno SC	0.0	T	0.0	T	0.0	T
Evidence 700 WG	0.0	T	0.0	T	0.0	T
Imidacloprid Nortox 480SC	22.87	T	21.64	T	12.84	T
MarshalStar EC	1.15	T	0.0	T	0.0	T
Regent 800 WG	37.24	T	27.17	T	6.65	T
SingularBR SC	52.29	MT	70.81	C	73.21	C
Talisman EC	2.73	T	2.98	T	0.00	T

a C: Compatible, T: Toxic, MT: moderately toxic.

It should be emphasized that the methodology used in this study does not allow its data to be fully extrapolated to a field situation where the tank mixture is exposed to abiotic factors, such as temperature and humidity, which can affect its efficiency. Another aspect that should be considered is that this study used conidia from a strain that was not protected by coformulants. The coformulants provide abiotic and biotic protection for microorganisms; therefore, the damaging effect of the pesticide when mixed with the biopesticide tends to be less than that when using a strain alone. This strain was chosen because many sugar cane mills produce their own *B. bassiana* by applying pure conidia or by mixing with mineral oil.^45^

In vitro methodology to assess the compatibility of entomopathogen strains with pesticides is advantageous because the pathogen is exposed to all the toxic effects of pesticides, eliminating unpredictable environmental field conditions.^13^ Moreover, this study provides information that can be used for applications associated with entomopathogens and pesticides, promoting increased susceptibility of the target insect as some synthetic substances cause stressful effects, facilitating infection and rapid proliferation of the disease caused by the pathogen.

The lack of standardization of the methods used to assess these effects on entomopathogens makes it challenging to compare the results of the same product. The conclusions reached in various studies show great diversity between them, with results that vary considerably, as they depend on the strain and species of fungus, their combination with different products, the concentrations of the active ingredients, and the formulation of each product tested. Given these factors, each isolate, fungal species, and product can show specific compatibility or incompatibility.^2^
